# Effectiveness of Self-Tennis Ball Massage Therapy on Low Back Pain Among Geriatric Individuals

**DOI:** 10.7759/cureus.78467

**Published:** 2025-02-03

**Authors:** Satish N Salvi, Nirmala A Londhe

**Affiliations:** 1 Community Health Nursing, Bharati Vidyapeeth (Deemed to be University) College of Nursing, Sangli, IND

**Keywords:** effectiveness, geriatric, low back pain, tennis ball, therapy

## Abstract

Introduction

Pain is a physiological and psychological response. For many people, it is a major problem that causes unpleasantness or aversion and reduces productivity in life. According to the International Association for the Study of Pain (IASP), pain is defined as “an unpleasant sensory and emotional experience associated with actual or potential tissue damage or described in terms of such damage". Low back pain is commonly faced by each individual. Nearly everyone is affected by it at some point. A staggering 619 million people worldwide suffered from low back pain in 2020 (nearly 10% of the world's population), and by 2050, that number is expected to reach 843 million. The treatment options for low back pain are the same all over the world such as applying muscle relaxant ointments or sprays, resting the back for a while, avoiding heavy physical activities for a couple of days, applying heat or cold compress, etc.

Objective

The primary objective is to evaluate the effectiveness of self-administered tennis ball massage therapy for low back pain. There are many benefits of massage therapy that show effective results. As an accepted part of many physical rehabilitation programs, massage therapy has also proven effective results for many chronic conditions, including low back pain, arthritis, bursitis, and fatigue. Low back pain is a common health issue that will affect eight out of 10 adults at some point in their lives. Although back pain is common, there are numerous effective methods to reduce muscle tension and provide relief.

Methodology

Pretest-posttest control group design was used, which included community-dwelling elderly individuals aged 60 years and above. Fifty participants in the experimental group and 50 in the control group were included in the present study. Ethical approval was obtained from the Institutional Ethical Committee. The researchers conducted a survey in the community area and participants were included as per ageing factors for those experiencing back pain. Individuals with severe cognitive impairment, contraindications to massage therapy, having gone through a surgical procedure and long-term bedridden were excluded. Pain scores were assessed using the WHO Numerical Pain Rating Scale (0: No pain, 1-3: Mild pain, 4-6: Moderate pain, 7-10: Severe pain) (see Appendices). In the primary stage pretest pain assessment was done and the next day onwards tennis ball massage was administered to the experimental group of geriatric individuals. On the sixth day, the posttest pain assessment was done.

Result

On day 1 (pre-test), before giving tennis ball massage therapy on low back pain, mean = 6.34, S.D. = 0.9606 in the experimental group. On day 6 (post-test), after giving tennis ball massage therapy on low back pain, mean = 2.84, S.D. = 0.8417 in the experimental group, and t = 23.9505 and p = 0.00001. Hence the result shows that the tennis ball massage is effective in reducing low back pain.

Conclusion

Since the test is statistically significant according to the pre-test and post-test in the experimental group at the 5% level of significance, the null hypothesis is rejected because the p-value for this comparison is smaller than 0.05. This indicates that self-administered tennis ball massage therapy is significantly effective for lower back pain.

## Introduction

Pain is a physiological and psychological response. For many people, it is a major problem that causes experience of unpleasantness or aversion and reduces the routine work experience. According to the International Association for the Study of Pain (IASP), pain is defined as “an unpleasant sensory and emotional experience associated with actual or potential tissue damage or described in terms of such damage" [1]. Low back pain is a universal health issue faced by each individual. Nearly everyone is suffered from it at some point in their lives. Pain can be classified into two types: acute or chronic. According to the medical definition from the Diagnostic and Statistical Manual of Mental Disorder (DSM) Fifth Edition, low back pain is a symptom related to acute or chronic pain in the lumbar or sacral regions and may be associated with sprains and strains of muscles and ligaments, displacement of the intervertebral disc, and other conditions. Acute back pain continues between four and 12 weeks, while chronic back pain persists for 12 or more weeks. The treatment options for low back pain are available in a variety of forms all over the world but it's dependent upon availability and cost-effectiveness [2,3]. There are pharmacological and non-pharmacological treatments proposed for this condition, and among them, massage therapy is considered a helpful treatment, offering benefits such as improvements in mobility and stress reduction. As per the clinical practice guidelines from the American College of Physicians and the American Pain Society massage therapy is one of the oldest healing arts and tennis ball massage therapy is effective to reduce low back pain and increase physical movements.

The benefits of massage therapy are to reduce low back pain and show effective physical movements. As an accepted part of many physical rehabilitation programs, massage therapy has also proven effective results for many chronic conditions, including low back pain, arthritis, bursitis, and fatigue. Low back pain is a common health issue that will affect eight out of 10 adults at some point in their lives [4]. Although back pain is common, there are so many effective ways to reduce muscle tension and provide relief. One of the easiest, most cost-effective, and convenient methods is self-tennis ball massage therapy [5]. Back pain prevalence and risk factors are notable in the U.S., which has the greatest incidence rates of low back pain (35%), arthritis (25%), and obesity (40%), all of which are significant contributing factors to chronic pain. About 1% to 37% of chronic low back pain patients may have a neuropathic component related to it [6]. Low back pain (LBP) is an emerging health problem globally, with middle-aged people (35-58 years) being particularly prone to it. In fact, 31 million Americans experience low-back pain at some point in their lives because of their occupational factors affected and not using ergonomics techniques while performing physical activity [7].

Dietary considerations

Adopting a Mediterranean diet or eating a plant-based diet - decreasing pro-inflammatory foods and adding anti-inflammatory foods to your diet - often helps reduce inflammation in the spine and joints. Non-vegetarian diet refers to meat, chicken, eggs, and fish. A mixed diet refers to a diet that includes fruits, vegetables, dairy products, grains and meat.

## Materials and methods

Pretest-posttest control group design was used, which included community-dwelling elderly individuals aged 60 years and above. Ethical approval was obtained from the Institutional Ethical Committee. Researchers conducted the survey in the community area like home-to-home survey and participants were screened according to the inclusion criteria, and those experiencing back pain were included in the study. Individuals with severe cognitive impairment, contraindications to massage therapy, having gone through a surgical procedure and long-term bedridden were excluded. Written informed consent was obtained from all participants. Pain scores were assessed using the WHO Numerical Pain Rating Scale (0: No pain, 1-3: Mild pain, 4-6: Moderate pain, 7-10: Severe pain).

Study participants

Participants were assigned to group A (Experimental group) and group B (Control group) using a purposive sampling technique for those individuals who faced low back pain. Assessment of pretest pain was carried out before starting the intervention and at the end of the sixth day, the posttest pain assessment was done. WHO Numerical Pain Rating Scale was used to evaluate the pain level. In the experimental group, geriatric individuals received tennis ball massage for 5 minutes per day with special instruction regarding self-tennis ball massage therapy used under the family members' supervision. Follow-up assessments were conducted on the sixth-day post-intervention.

Tennis ball massage technique

Tennis ball massage therapy is very cost-effective, easily available and easy to use as compared to other available methods. Place the tennis ball on the ground and then lie on it in the supine position, keeping the knees bent. The tennis ball should be parallel to the waist and centered just above the lower back. The tennis ball should be situated in a way that it contacts the muscle tissue on each side of the spinal column. Adjust yourself until it feels balanced and comfortable and then raise both arms with fingers pointed towards the ceiling. Keep the arms as straight as possible. Beginning with the right or left arm, slowly lower the arm back toward the head. While keeping the arm straight feel free to bend the neck backward when lowering the arm. Hold this position for a few seconds, then slowly bring it back to its original starting position.

Intervention

In the primary stage, the researchers showed the tennis ball massage technique to geriatric individuals and other family members. The researchers had learned about the technique of giving tennis ball massage therapy from the physiotherapy department. The tennis ball was purchased from the market. As per the review of the literature, the researchers decided the duration of the intervention to be a total of six days. During this period, the respective experimental group participants received tennis ball massage therapy for 5 minutes. The control group participants did not receive any tennis ball massage therapy, and they continued with their routine treatment. The assessment was taken pre-intervention and post-intervention for both the groups.

Sample selection criteria

The participants were selected as per inclusion criteria, i.e., geriatric individuals aged 60 years and above, and individuals who were willing to participate. The participants were excluded from the study as per exclusion criteria, i.e., individuals with severe cognitive impairment assessed by a mental status questionnaire, individuals with contraindications to massage therapy, and individuals with any operative procedure and bedridden for a long time.

Ethical consideration

The Institutional Ethical Committee Bharati Vidyapeeth (Deemed to be University) College of Nursing (IECBVDUCON, Sangli, Maharashtra, India; no. EC/NEW/INST/2024/MH/0414) approved the study as “Effectiveness of Self-Tennis Ball Massage Therapy on Low Back Pain Among Geriatric Individuals.”

Statistical analysis

For data analysis, IBM SPSS Statistics 26.0 (IBM Corp., Armonk, NY, USA) was used. Descriptive statistics provided insights into the mean and standard deviation for each group. As per the objectives, the Chi-square test was used to analyze the data, and the paired t-test was used to compare pre-test and post-test results between the experimental and control groups. All statistical tests were performed with a significance threshold set at p<0.05.

## Results

In the experimental group, 62% were in the age group of 60-65 years, 30% in the age group of 66-70 years and 8% in the age group of 70 years and above. In the control group, 54% were in the age group of 60-65 years, 28% in the age group of 66-70 years and 18% in the age group of 70 years and above. In the experimental group, 60% of the participants were male and 34% were female. In the control group, 36% of participants were male and 64% female. In the experimental group, 18% were vegetarian, 60% were non-vegetarian and 22% had a mixed diet. In the control group, among the 50 participants, 24% were vegetarian, 34% non-vegetarian and 42% had a mixed diet. The frequency and percentage distribution of demographic data are shown in Table 1.

**Table 1:** Frequency and percentage distribution of demographical variables

Sr.No	Demographic Variables		Experimental Group		Control Group	
			Frequency	Percentage (%)	Frequency	Percentage (%)
1.	Age (in years)	60-65	31	62	27	54
		66-70	15	30	14	28
		71 & above	04	08	9	18
2.	Gender	Male	33	66	18	36
		Female	17	34	32	64
3.	Diet	Vegetarian	9	18	12	24
		Non-Vegetarian	30	60	17	34
		Mixed	11	22	21	42

By observing the level of pain before giving self-tennis ball massage in the experimental and control groups, the following results were obtained: On day 1, 56% of participants in the experimental group and 54% in the control group had a moderate level of pain before giving self-tennis ball massage. There were 44% in the experimental group and 46% in the control group who had a severe level of pain before giving self-tennis ball massage. So, the above statistical values show that geriatric people above 60 years of age had common health issues like low back pain. Assessment of the level of low back pain before giving self-tennis ball massage among geriatric individuals in the experimental and control groups is shown in Table 2.

**Table 2:** Assessment of level of low back pain before giving self-tennis ball massage among geriatric individuals in the experimental and control groups

Pre-Test		Experimental Group		Control Group	
		Frequency	Percentage (%)	Frequency	Percentage (%)
Pain Score	Mild (0-3)	00	00	00	00
	Moderate (4-6)	28	56	27	54
	Severe (7-10)	22	44	23	46

By observing the level of pain after giving self-tennis ball massage in the experimental and control groups following results were obtained: On day 6, 82% of participants in the experimental group and 0% in the control group had mild levels of pain after giving self-tennis ball massage. There were 18% in the experimental group and 54% in the control group who had a moderate level of pain after giving self-tennis ball massage. There were 0% in the experimental group and 46% in the control group who had a severe level of pain after giving self-tennis ball massage. So, it is proved that tennis ball massage is more effective to minimize the low back pain. Assessment of the level of low back pain after giving self-tennis ball massage among geriatric individuals in the experimental and control groups is shown in Table 3.

**Table 3:** Assessment of the level of low back pain after giving self-tennis ball massage among geriatric individuals in the experimental and control group

Post-Test		Experimental Group		Control Group	
		Frequency	Percentage (%)	Frequency	Percentage (%)
Pain Score	Mild (0-3)	41	82	00	00
	Moderate (4-6)	09	18	27	54
	Severe (7-10)	00	00	23	46

On day 6, after giving tennis ball massage therapy on low back pain, mean = 2.84, S.D. = 0.8417 in the experimental group and in the control group mean = 6.36, S.D. = 0.9847, t = 19.2125 and p = 0.00001. Hence the result shows that it is significant. So, the above-mentioned statistical values proved that tennis ball massage therapy was effective in the experimental group. A comparison of the post-test after giving tennis ball massage therapy in the experimental and control groups is shown in Table 4.

**Table 4:** Comparison of post-test after giving tennis ball message therapy in the experimental and control groups.

Post-test	Mean	S.D.	t- value	p-value	Significance
Experimental Group	2.84	0.8417	19.2125	0.00001 < 0.05	Significant
Control Group	6.36	0.9847			

On day 1 (pre-test), before giving tennis ball massage therapy on low back pain, mean = 6.34, S.D. = 0.9606 in the experimental group. On day 6 (post-test), after giving tennis ball massage therapy on low back pain, mean = 2.84, S.D. = 0.8417 in the experimental group and t = 23.9505 and p = 0.00001. Hence the result shows that the tennis ball massage is effective to reduce low back pain. A comparison of the pre-test and post-test after giving tennis ball massage therapy in the experimental group is shown in Table 5.

**Table 5:** Comparison of pre-test and post-test after giving tennis ball message therapy in the experimental group

Experimental Group	Mean	S.D.	t-value	p-value	Significance
Pre-test	6.34	0.9606	23.9095	0.00001 < 0.05	Significant
Post-test	2.84	0.8417			

## Discussion

There are few similar studies conducted, and their results are also similar to the present study. A total of 7669 adult (aged 18 to 75 years) participants were included in the study, and they were given treatment as prescribed by the physician, as well as tennis ball massage therapy. The questionnaire used included a pain symptoms history to identify the site of any pain, and it was mailed to the entire study population, with two repeat mailings sent to non-responders. The care provided by medical professionals for low back pain to individuals from the study population was monitored for 12 months using computerized records of all surgery contacts. Of the study population, 4,501 (59%) responded. The one-month period prevalence of low back pain was 39% (35% in males, 42% in females). The age distribution was unimodal, with the peak prevalence of low back pain in those aged 45 to 59 years. Responders to the first mailing had a small but non-significant increase in prevalence compared to those who responded to the second or third mailing. Non-responders had a subsequent consultation rate for low back pain that was 22% lower than that for survey responders [8]. In the present study, the researchers used a quasi-experimental pretest-posttest control group design. A nonprobability purposive sampling technique was adopted with 100 participants - 50 in the experimental group and 50 in the control group. Ethical clearance was obtained. Data was analyzed using descriptive and inferential statistics. Association was determined using Fisher's exact test, and a paired t-test was used to assess the effect of self-tennis ball massage therapy on low back pain among patients.

Results

After the massage therapy, it was found that 30% of the participants had mild pain, and 70% had moderate pain. On day 4, the second observation after the intervention, 93.3% of participants in the experimental group had mild pain and 6.7% had moderate pain. In the control group, 36.7% of participants had moderate pain and 63.3% had severe pain. On day 4, in the control group, 10% of participants had moderate pain and 90% had severe pain [9].

## Conclusions

In the present study, the researchers used tennis ball massage therapy to assess the low back pain. There were also different methods available to reduce low back pain but as compared to other methods the tennis ball massage therapy was cost-effective, easily available and easy to handle, and it increases productivity in life. The present study result shows that the tennis ball massage therapy was effective. The study reveals a statistically significant difference between the experimental and control groups in post-test levels of low back pain among geriatric individuals. The results demonstrate that tennis-ball massage therapy significantly reduces low back pain in this population. This suggests that tennis-ball massage therapy is an effective intervention for alleviating low back pain in geriatric individuals.

## Figures and Tables

**Figure 1:**
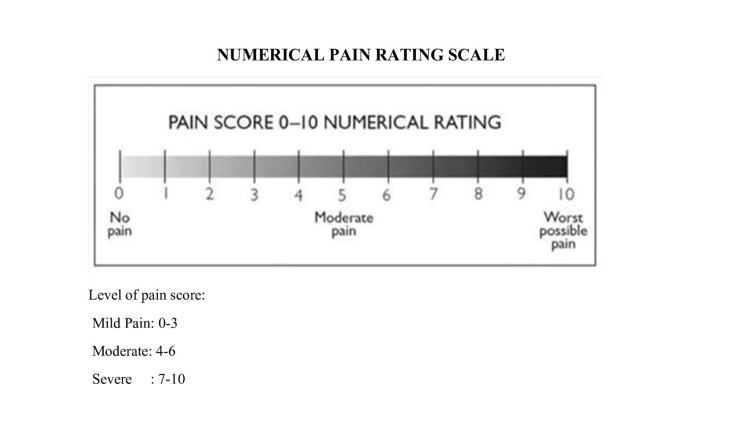
Numerical Pain Rating Scale

**Figure 2:**
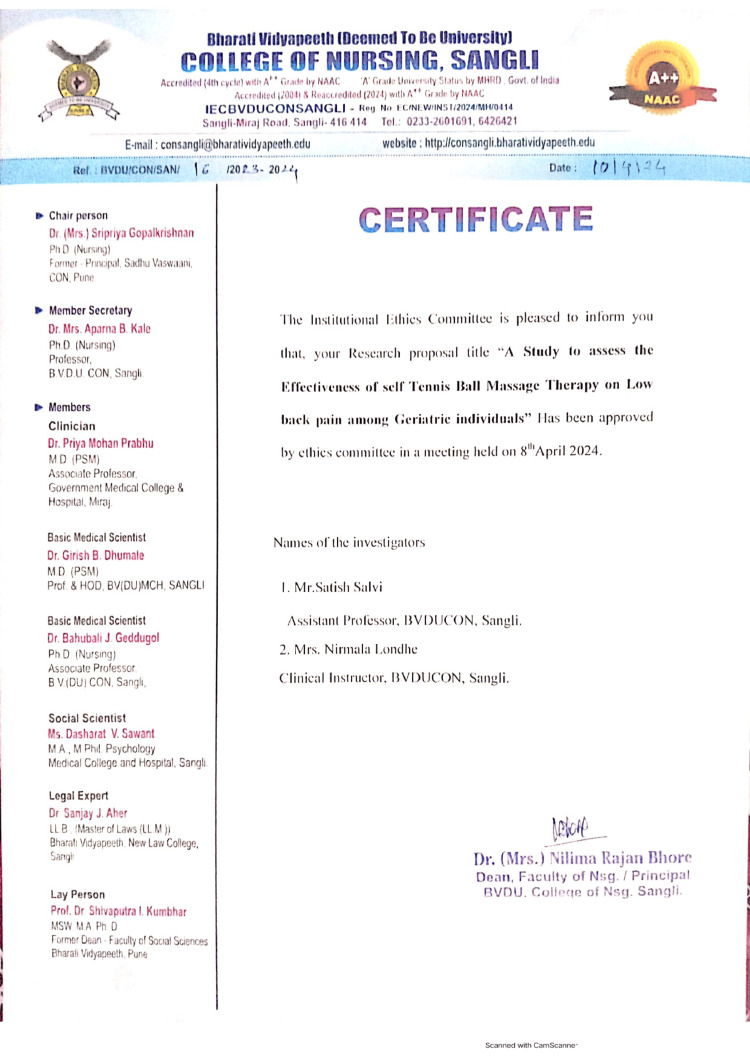
Ethical Approved Letter
